# Abnormal Foot Progression Angle Kinematics in Cervical Dystonia Improved After Osteopathic Manipulative Medicine: A Prospective Case Series

**DOI:** 10.7759/cureus.26459

**Published:** 2022-06-30

**Authors:** Jayme Mancini, Zachary Oliff, Reem Abu-Sbaih, Joseph Simone, Andrea LaRosa, Sonu Mody, To Shan Li, Adena Leder

**Affiliations:** 1 Osteopathic Manipulative Medicine, New York Institute of Technology (NYIT) College of Osteopathic Medicine, Old Westbury, USA; 2 Osteopathic Manipulative medicine, New York Institute of Technology (NYIT) College of Osteopathic Medicine, Old Westbury, USA; 3 Osteopathic manipulative medicine, New York Institute of Technology (NYIT) College of Osteopathic Medicine, Old Westbury, USA; 4 Medicine, New York Institute of Technology (NYIT) College of Osteopathic Medicine, Old Westbury, USA

**Keywords:** kinematics, chronic pain, cranial osteopathic manipulative medicine, manual-therapy, tremor dystonia, spasmodic torticollis, foot progression angle, gait, osteopathic manipulative treatment, cervical dystonia

## Abstract

Introduction

Cervical dystonia (CD), a rare disorder, is the most common form of dystonia, a movement disorder. Impairments in activities of daily living and quality of life may result from chronic pain, perceived stigma, difficulty walking, and/or lack of control over movements. Studies of treatments for difficulty walking in CD have been inconclusive. Osteopathic manipulative medicine (OMM) has been used to improve gait biomechanics in other health conditions. Foot progression angle (FPA) while walking indicates functional gait abnormalities that increase the risk of knee injury and osteoarthritis.

Objective

The aim of this study is to test if five-weekly treatments using an OMM sequence designed for CD improved abnormal gait biomechanics in individuals with CD by identifying and addressing somatic dysfunctions.

Methods

In this prospective case series, independently ambulating individuals with CD symptom onset before the age of 40 years, not due to traumatic injury, were evaluated utilizing validated scales for severity (Toronto western spasmodic torticollis rating scale [TWSTRsI]) and symptoms affecting quality of life (Cervical Dystonia Impact Profile [CDIP-58]), physical examination, and FPA before and after five-weekly OMM treatments. Lower body joint range of motion and angles were captured in a clinical gait lab by nine cameras collecting three-dimensional Whole-body position data during three trials of one gait cycle at participant-selected walking speed. The FPA waveforms during the gait cycle were quantified by Vicon Nexus and Polygon applications. Pretreatment and posttreatment results were compared to established healthy gait waveforms and tested by repeated measures ANOVA (α=0.05).

Results

Pretreatment waveforms in CD had a mean 5.13° of excess FPA during gait cycle phases requiring lower-extremity pronation compared to previously published age-gender-matched healthy waveforms. There was 96% improvement in pronation after five treatments, with a mean 0.21° (p=0.041) of excess FPA. Mean TWSTRs and CDIP-58 scores improved. On physical examination, the rotational direction of C2 vertebrae was contralateral to neck muscle hypertonicity. Vertical sphenobasilar synchondrosis strains were present in those with anterotorticollis. Participants had ipsilateral anterolateral neck muscle and anterolateral abdominal wall muscle hypertonicity. All patients had pelvic somatic dysfunctions with left-side superior relative to right-side and restriction from lower-extremity pronation (i.e., supination dysfunctions).

Conclusion

The FPA was significantly improved after treatment. This OMM sequence was well tolerated and may be useful for improving gait kinematics in individuals with CD. Randomized, controlled, long-term studies are needed to determine effectiveness.

## Introduction

Although cervical dystonia (CD), including spasmodic torticollis, is a rare disorder, it is one of the most common forms of dystonia seen by neurologists. Impairment in activities of daily living (ADL) and quality of life may result from chronic pain, perceived stigma of the disorder, limited range of motion (ROM) in the upper body joints, difficulty walking, and the lack of control over dystonic muscles [1,2]. Although the exact prevalence of CD is unknown, it occurred in approximately 0.390% of the United States in 2007 and had a worldwide prevalence of 57 per 1 million [3]. CD is characterized by involuntary muscle contraction in the neck, usually causing twisting movements or abnormal postures [1-3]. Painful consequences may include spinal degeneration, inflammation of the nerve roots, and frequent headaches. Individuals with CD self-rate having difficulty walking on the validated symptom and quality of life scale Cervical Dystonia Impact Profile (CDIP-58) [2,4,5]. Identifying and treating the factors contributing to abnormal gait biomechanics such as foot progression angle (FPA) may decrease the insidious impact it has on cardiac workload and osteoarthritis [5-11]. Previous studies suggested that osteopathic manipulative medicine (OMM) may improve gait biomechanics in non-CD individuals [11-13].

Clinical neurological and musculoskeletal examination studies found that CD adversely affects gait [4,5]. Hoffland et al. found a significantly slower walking speed and “reduced confidence in one’s own balance” in CD patients [4]. Although studies suggest that gait retraining with physical therapy provided a useful approach to reducing medial knee joint loads during gait, there has had inconsistent results on pain and progression of osteoarthritis [6,7,10]. During OMM, manual forces are directed to improve physiological function and homeostasis by reducing the joint tissue distortions, laxity, and restrictions in osteopathic somatic dysfunctions. Somatic dysfunctions are biophysical limitations from healthy function such as muscle length and strength imbalances, restrictions in joint range of motion and alignment, distortion of fascia, connective tissue weakness, and edema [11]. However, the relationship between somatic dysfunctions and motion analysis kinematics is unclear. A pre-defined sequence of OMM was developed based on prior retrospective case reports on CD, in which joints found to have severe somatic dysfunctions were identified [5,12], and through clinical decision-making experience in patient care based on the neurological, fascial, and muscular factors affecting gait patterns and balance in healthy individuals compared to those with neck pain or neuromuscular disorders [11,13-16]. Fascia is an integral part of the musculoskeletal system. It mediates myofascial force transmission and proprioceptive communication as well as creating pretension in musculature [17]. Observing the functional aspects of gait and balance can lead to a patient-centered treatment approach [15].

The FPA is used in the assessment of gait because it is indicative of functional abnormalities in knee loading and hip and pelvic muscles that increase the risk of knee injury and osteoarthritis. The FPA is the angle formed between the longitudinal axis of the foot and the forward line of progression during walking [18,19]. Specifically, an increase in the FPA increases the load on medial column support in the ipsilateral knee, which, in turn, leads to greater risk of injury to this compartment [19]. Repetitive joint injury frequently leads to osteoarthritis [6-9,18].

During healthy walking, the heel strikes the ground with the lateral border of the calcaneus [11]. Next, the fifth metatarsal contacts the ground followed by loading of the body weight onto all of the metatarsals [11]. As full contact of the foot with the ground ensues, the tibia rotates internally and the talar-calcaneal joint pronates (anteromedial glide of the talus over the everting calcaneus) [11]. The midtarsal joint, which consists of the talonavicular and calcaneal-cuboid articulations, unlocks with subtalar joint pronation. In pronation, the forefoot is more efficient in adapting to changes in ground terrain. During the midstance phase, the tibia externally rotates and the foot supinates (posterolateral glide of the talus over the inverting calcaneus) preparing for propulsive force exertion [11]. The midtarsal joints lock during subtalar joint supination, and the bones have efficient pull of peroneus longus and posterior tibialis muscles. Synergistic muscle contraction stabilizes the midfoot and alignment of the first metatarsal-phalangeal joint for push-off [20]. Restrictions to these joint motions or asynchronous movement patterns may be found in assessment of FPA when they prevent a healthy progression from pronation to supination [11].

Here, we tested the hypothesis that abnormal gait biomechanics in individuals with CD will improve with identification of somatic dysfunctions and treatment with a pre-defined OMM sequence.

## Materials and methods

Independently ambulating men and women with CD symptoms beginning before the age of 40 years not due to accident or acute injury were eligible for this IRB-approved (NYIT BHS983) prospective case series with repeated measures. Participants were recruited directly through their physicians.

The validated scales, Toronto western spasmodic torticollis rating scale (TWSTRsI) and CDIP-58, were used to determine the changes in disease sign and symptom severity before and after five-weekly OMM treatments. Symptoms of CD involve pulling, pain, or stiffness of the neck and associated head tilt [1-3]. The degrees of head tilt appear to affect severity. Tilt is categorized into directions of rotational torticollis: retrocollis, anterocollis, and laterocollis [1]. Severity may be quantified by TWSTRs. The CDIP-58 quantifies (a) symptoms involving the head and neck, pain and discomfort, and sleep; (b) daily activities involving upper limb activities and walking; and (c) psychosocial sequelae including annoyance, mood, and psychosocial functioning. The mean and standard deviations (±SD) were calculated.

FPA was assessed by a clinical gait lab using quantitative motion analysis of the whole-body Plug-in-Gait Vicon skeletal template ([Centennial, CO, USA]). The skeletal template includes 35 spherical markers of anatomical landmarks. Participants were instructed to walk as they normally would at a self-selected speed. One gait cycle was captured three times with nine Vicon MX series cameras before and after five-weekly 30-minute OMM treatments. Joint angles were quantified using Vicon Nexus 4.4 and Polygon 4.1. The FPA was calculated by the software as the angle between the foot vector (the line joining the ankle joint center and the marker over the second metatarsal head) and the forward laboratory axis.

The pre-defined sequence of OMM in Table 1 was developed based on prior case reports of CD in which joints found to have severe somatic dysfunctions were identified [5,12]. Physical examinations and OMM were performed in five-weekly sessions in an academic health care center on each subject by board-certified physicians. The OMM procedure was applied to somatic dysfunctions to improve muscle tone balance and joint alignment of the feet, knees, pelvis, sacrum, spine, respiratory diaphragm, ribs, abdominal musculature, head, neck, and shoulders. The OMM techniques used were balanced-ligamentous-tension, cranial, muscle energy, myofascial release, and Still’s technique [11].

**Table 1:** Steps in the OMM sequence utilized weekly for cervical dystonia OMM, osteopathic manipulative medicine

Step #	Treatment description
1	Indirect suboccipital myofascial release
2	Balanced tension of occiput (fourth and fifth fingers), occipital condyles (third fingers), temporal bones (thenar eminence), and C2 (index fingers)
3	Direct cranial OMM of sphenobasilar synchondrosis using vault hold
4	Oculocephalogyric reflex muscle energy for cervical spine segmental somatic dysfunctions using isometric contraction for 15 seconds for each of three repetitions
5	Decompression of occipitomastoid and sphenofrontal sutures
6	Ligamentous articular strain technique for hypertonic anterior and lateral cervical fascia or eccentric contraction muscle energy for spasmodic sternocleidomastoid and scalene muscles
7	Muscle energy for hyoid deviation
8	Myofascial release of superior thoracic aperture
9	Myofascial release of rectus abdominis muscles and substernal fascia
10	Balance ligamentous tension of thorax with hands on T3-7 and sternum
11	Respiratory muscle energy of the thoracolumbar diaphragm
12	Muscle energy for innominate dysfunctions and sacral dysfunctions
13	Still’s technique for femur-tibia and tibia-fibula dysfunctions
14	Indirect balanced ligamentous tension of navicular dysfunctions
15	Whole-body muscle energy for side-bending dysfunction on the side of greater neck muscle hypertonicity using isometric contraction for 15 seconds for each of three repetitions

The mean left and right FPA of 3 gait cycles at 0%, 20%, 40%, 60%, 80%, and 100% of the gait cycle were calculated for study participants and compared to age- and gender-matched FPA previously determined to be normal, healthy kinematic waveforms at these gait intervals [16] to determine pre- and post-treatment severity. Significant changes in FPA waveforms pre- to post-treatment during the gait cycle were tested by repeated measures ANOVA (α=0.05).

## Results

Participants meeting eligibility criteria and providing informed consent were all Caucasian females (n=6) aged 25-67 years with healthy body mass indexes ranging from 19.1 to 25.8. There were five participants who withdrew from the study due to time commitment. The number of years of having dystonic symptoms ranged from 9 to 48 years. The mean (n=6) TWSTRs score trended 10% (±8) toward improvement. There was a mean improvement of 10% (±4) in the CDIP-58 total transformed score, with the greatest change on the pain and discomfort subscale of 18% (±10).

Physical examination

The participants’ direction of the head from neutral was anterotorticollis in four, torticollis in one, and retrocollis in one. Additional features were position-dependent dystonic head tremor in four participants and spasmodic dysphonia of vocal cords in one participant. The pertinent physical examination findings treated with OMM are depicted in Table 2. On palpation, there was hypertonic musculature over the occipitomastoid suture(s) on the more affected side of the torticollis. Also, a decreased osteopathic cranial rhythmic impulse was found [21] in the temporal bone on the affected side or side of the torticollis. The strain patterns of the sphenobasilar synchondrosis were vertical [22] in the presence of anterocollis and retrocollis, and torsional strains were present in all participants. Of note, the participant with right torticollis since birth also had moderate plagiocephaly. There was severe restriction to the range of motion of the cervical vertebrae 2, the sacroiliac joints, and lower extremity pronation-supination in all participants. In addition, iliac crest asymmetry was present in all participants. There were no adverse events. The OMM intervention was well tolerated.

**Table 2:** Osteopathic physical examination findings of CD patients SBS is the junction between the sphenoid and the occiput. A torsional strain of the SBS is when the sphenoid and occiput are slightly rotated in opposite directions about an anterior to posterior axis through the SBS. A vertical strain of the SBS is when the sphenoid and occiput have rotated in the same direction about two parallel transverse/horizontal axes through the sphenoid and occiput. A lateral strain of the SBS is when the occiput and sphenoid have rotated around two parallel superior-inferior axes. Lateral and vertical strains are non-physiologic and likely a result of trauma or other health conditions [11,22]. *Out-rotation is equivalent to lateral rotation here CD, cervical dystonia; SBS, sphenobasilar synchondrosis; SD, osteopathic somatic dysfunction; WNL, within normal limits

Age (years)	63	34	67	32	65
Age at onset of CD (years)	19	25	29	Birth	13
Directions of head tilt or shift	Right anterotorticollis	Right anterotorticollis	Right anterotorticollis (Figure 1)	Right torticollis	Retrocollis
Additional features	Dystonic tremor	Laryngeal dystonia	Dystonic tremor	Moderate plagiocephaly	Dystonic tremor
SBS strain pattern	Superior vertical right torsion	Superior vertical right torsion	Superior vertical right torsion	Left lateral right torsion	Inferior vertical left torsion
C2 SD	Flexed, rotated & side-bent to the left	Flexed, rotated, and side-bent to the left	Flexed, rotated, and side-bent to the left	Flexed, rotated, and side-bent to the right	Extended, rotated, and side-bent to the right
Innominate SD	Right anterior innominate rotation, inflared	Right anterior innominate rotation	Right anterior innominate rotation, inflared	Right anterior innominate rotation	Left superior shear
Tibial torsion SD*	Bilateral out-rotation	Bilateral out-rotation	Right out-rotation	WNL	Bilateral out-rotation
Fibular head SD	Posterior bilaterally	WNL	WNL	WNL	WNL
Talus SD	WNL	Right plantar-flexed	WNL	WNL	Bilateral plantar flexed
Calcaneous SD	Inverted bilaterally	Right inverted	Inverted, subluxed bilaterally	Inverted bilaterally	Inverted bilaterally
Navicular SD*, cuboid SD	Right out-rotation	Bilateral out-rotation	Bilateral dropped cuboid	WNL	Bilateral lateral rotation/ supination

Before OMM, the mean number of degrees of FPA abnormality at 0%, 20%, 40%, 60%, 80%, and 100% of one gait cycle per participant was 5.13 degrees (n=5). Participants had worse FPA, or greater degrees of abnormal FPA, during the phases of the gait cycle in which lower extremity pronation is required for healthy joint kinematics. The same measures showed significant improvement after five-weekly treatments, with a mean 0.21° (p=0.041) beyond healthy waveforms.

## Discussion

The results of this prospective, uncontrolled case series of OMM targeted for improving walking and balance in CD are consistent with previous studies suggesting that individuals with CD have the signs and symptoms on the disease-specific scales TWSTRs and CDIP-58, including difficulty walking and chronic neck and/or shoulder pain [1-3]. CD can also affect the small muscles involved in visuotemporal coordination, vocalization, swallowing, and upper airway control, and sometimes impacts balance [2,4]. Previous studies on chronic neck pain of variable etiology revealed that inter-regional spinal kinematics were poorly coordinated [16], including significantly different muscle recruitment and firing sequence during functional tasks compared to healthy controls [15,16]. Although the sphenobasilar synchondrosis fuses during adolescence and ossifies by the third decade of life [23, 24], the small amplitude of motion here continues into adulthood because of the viscoelastic and viscoplastic properties of the bones in conjunction with the sutures [25].

This study was limited by the small sample size of participants with this rare disorder, variable subtypes of CD, and lack of control participants. Although a control group was approved by the IRB, recruited participants in the control group did not continue to participate in the study due to the time commitment and the risk of social embarrassment of anyone focusing on their walking without the benefit of the OMM intervention. Quantitative motion analysis of gait may be used to assess the effectiveness of OMM in improving gait in CD [18].

The effects of CD on other regions of the body may be either a strictly biomechanical compensation [10,14-16] and/or a neural network response such as reflexes secondary to nociception or joint derangement [17]. The downstream effects of this movement disorder appear to involve abnormal FPA. The detailed physical examination utilized to determine somatic dysfunctions to apply the OMM sequence given in Table 1 once weekly for five weeks significantly improved FPA and made improvements in participants’ self-reported symptoms affecting quality of life. This intervention is noninvasive and may be used in combination with other treatment approaches. Further research is necessary to determine the relationship between whole-body motion analysis and somatic dysfunction biomechanics of CD contributing to an abnormal FPA. The long-term effects of this intervention were not assessed.

## Conclusions

In this prospective study of a pre-defined OMM treatment for gait biomechanics in individuals with CD, the observations identified somatic dysfunctions that appear to contribute to disease severity and/or abnormal FPAs, which affect walking. The rotation direction of C2 was opposite the side of neck muscle hypertonicity, and vertical strains were present in those with anterotorticollis. Participants had anterior neck muscle and anterior abdominal wall muscle hypertonicity on the same side. All participants had pelvic somatic dysfunctions with the left side superiorly relative to the right side (i.e., left superior shear, right inferior pubic symphysis, right anterior rotation dysfunctions) with bilateral restrictions to lower extremity pronation. The hips were also restricted from healthy ranges of rotation. Hip rotation was opposite the direction of innominate flare dysfunction. The effects on acetabular angles and the hip joint over time are unclear. Participants had worse FPA during the phases of the gait cycle in which full contact of the foot with the ground is made; when lower extremity joints are restricted from pronation, the tibia is unable to rotate internally and the talar-calcaneal joint is unable to pronate, preventing the natural anteromedial glide of the talus over the everting calcaneus. The abnormal FPA demonstrated in CD participants indicates abnormal loading on the hip flexor muscles and other muscles in the pelvis and hips, which warrants further study. The significantly improved FPA in CD suggests that OMM may be used to increase support during the double support phase and to increase single limb support when the opposite limb is in advanced swing. The treatment was well tolerated and safe. However, further research is necessary to correlate whole-body motion analysis and somatic dysfunction biomechanics. Randomized, controlled, long-term studies are needed to test the hypothesis that treatment of these biomechanical dysfunctions with OMM is effective for unhealthy FPA in individuals with CD.
